# Quality Assurance of a Cross-Border and Sub-Specialized Teleradiology Service

**DOI:** 10.3390/healthcare10061001

**Published:** 2022-05-28

**Authors:** Szabolcs Hetenyi, Leonie Goelz, Alexander Boehmcker, Carlos Schorlemmer

**Affiliations:** 1 European Telemedicine Clinic SL, Torre Mapfre, C/Marina 16-18, 08005 Barcelona, Spain; shetenyi@telemedicineclinic.com (S.H.); alexanderb@aidoc.com (A.B.); cschorlemmer@telemedicineclinic.com (C.S.); 2 Department of Radiology and Neuroradiology, BG Klinikum Unfallkrankenhaus Berlin, Warener Straße 7, 12683 Berlin, Germany; 3 Institute for Diagnostic Radiology and Neuroradiology, University Medicine Greifswald, Ferdinand-Sauerbruch-Straße, 17475 Greifswald, Germany; 4 AIDOC Medical, Aminadav St. 3, Tel Aviv-Yafo 6706703, Israel

**Keywords:** teleradiology, sub-specialization, subspecialization, quality assurance, project report

## Abstract

Background: The current literature discusses aspects of quality assurance (QA) and sub-specialization. However, the challenges of these topics in a teleradiology network have been less explored. In a project report, we aimed to review the development and enforcement of sub-specialized radiology at Telemedicine Clinic (TMC), one of the largest teleradiology providers in Europe, and to describe each step of its QA. Evaluation: The company-specific background was provided by the co-authors—current and former staff members of TMC. Detailed descriptions of the structures of sub-specialization and QA at TMC are provided. Exemplary quantitative evaluation of caseloads and disagreement rates of secondary reviews are illustrated. Description of Sub-specialization and Quality Assurance at TMC: Sub-specialization at TMC is divided into musculoskeletal radiology, neuroradiology, head and neck, a body, and an emergency section operating at local daytime in Europe and Australia. Quality assurance is based on a strict selection process of radiologists, specific reporting guidelines, feedback through the secondary reading of 100% of all radiology reports for new starters, and a minimum of 5% of radiology reports on a continuous basis for all other radiologists, knowledge sharing activities and ongoing training. The level of sub-specialization of each radiologist is monitored continuously on an individual basis in detail. After prospective secondary readings, the mean disagreement rate at TMC indicating at least possibly clinically relevant findings was 4% in 2021. Conclusion: With continuing and current developments in radiology in mind, the essential features of sub-specialization and innovative QA are relevant for further expansion of teleradiology services and for most radiology departments worldwide to respond to the increasing demand for value-based radiology.

## 1. Introduction

### 1.1. Teleradiology and Sub-Specialization—Current Needs in Radiology?

The absolute number and distribution of specialized personnel has long been identified as a crucial issue in everyday medical practice [1,2]. The global shortage of radiologists has resulted in multiple urgent reports and even distress notifications by the medical community [3,4,5,6,7]. In the United Kingdom, 58% of clinical directors stated that they have too few radiology consultants “to deliver safe and effective patient care” [8]. The need to process growing numbers of ever more complex imaging studies has significantly affected the workload of practicing radiologists [9]. Teleradiology services all over the world are increasingly addressing these issues [10]. Balancing workloads among radiologists is only one benefit of teleradiology. Teleradiology networks with a high volume of cases can also select the most experienced radiologist for a certain imaging study [11].

Sub-specialization in medicine is a natural process after which an experienced specialist is dedicated to only one organ or one organ system [12]. At the turn of the millennium, 28% of radiologists in the U.S.A. were sub-specialized, whereas in 2018 45% of radiologists were working with some degree of specialization [13,14]. One reason for sub-specialization is evidence in the literature that disagreement rates between primary reporters and radiologists at tertiary referral centers can reach up to 42% in adult and pediatric populations [15]. The growing level of detail and complexity of imaging studies contributes to this divergence. Additionally, some authors have reported reduced turn-around times (TATs) for sub-specialized radiologists when working in their respective fields [16,17]. Evaluating the need for sub-specialization in radiology is, therefore, a discussion about quality as well as quantity [12].

### 1.2. Quality Assurance in (Tele-) Radiology

Quality assurance (QA) is the task of securing a certain standard of a product through appropriate measures [18]. In the past, quality concerns in radiology and teleradiology have often circled around image quality, image transfer, and dose management [19,20,21]. Other authors have focused on process management, certification, and economization [22,23]. In parallel, radiologists also began to demand “professional quality” in addition to technical QA [24,25]. Approaches to improve report quality are manifold. Some radiological institutions solely rely on multidisciplinary team meetings (MDTM) as a form of peer review [26]. During image preparation and presentation, radiologists have a chance to review a colleague’s report retrospectively and generate their own assessment of a certain case. It is essential that peer review/feedback reaches the primary reporting radiologist directly and that addendums are included in the radiology report. In an attempt to avoid information loss during peer-review, standardized processes have been developed as simple as the implementation of an email template for feedback [27]. The Royal College of Radiologists (RCR) defines a goal of performing peer review on 5% of studies as an “indicator of good reporting practice” [28]. Other groups have established web-based tools [29] and even workstation-integrated programs for retrospective and prospective peer-review [30].

### 1.3. Brief History of Telemedicine Clinic

The Telemedicine Clinic was founded in 2002 in Barcelona by a Swedish team of visionaries to “make the world’s best competence available to the smallest hospital in Europe” [31]. Radiology reports are provided by specialized radiology consultants. During the day, elective imaging reports are provided mainly for magnetic resonance imaging (MRI), computed tomography (CT), and conventional radiography (CR) from Barcelona and other parts of Europe. During the European night, radiologists accredited in Europe but based in Australian offices take over and provide emergency teleradiology services for the European customers.

Another significant aspect of the company is its IT department. Seventeen computer experts and 16 software developers assure smooth workflows and provide constant innovation. The development of a unique radiology information system (RIS) called Optemis (TMC, Barcelona, Spain) by this team contributed greatly to the success of TMC. The custom-made RIS paved the way for a unique platform in which imaging cases are distributed to the most qualified specialists. The software also enables automated assignment of cases for second reading and communication between radiologists during the peer-review and peer-feedback process. The IT experts also maintain the commercially available picture archiving system (PACS) from Sectra (Sectra HQ, Linköping, Sweden) used at TMC. Starting with one customer in 2003 TMC has evolved into one of the largest private teleradiology services in Europe, with 14 customers in Denmark, 24 in Sweden, and 46 customers in the UK. In total, 150 facilities are serviced by TMC. To date, TMC is active in the United Kingdom (UK), Sweden, and Denmark (DK). The main office is still located in Barcelona, and other sites are in Reading (UK), Sydney and Noosa (Australia), as well as Copenhagen (DK). In 2021 the workload was mastered by 216 elective radiologists and 79 emergency radiologists in full- and part-time. To date, we have not reported on the detailed aspects of TMC’s cross-border teleradiology network [32].

### 1.4. Intention

The current literature discusses aspects of QA and sub-specialization. However, the challenges of these issues in a teleradiology network have been less explored.

The aim of this project report was to review the development and enforcement of sub-specialized radiology at TMC as well as to illustrate each step of QA at this private teleradiology service. The focus of this inquiry will lie in the elective teleradiology service of TMC.

## 2. Evaluation

The company-specific background was provided by the co-authors—current and former staff members of TMC. Their insights were collected through a thorough discussion of strategy and facts. The backbone of this report is the detailed description of the structures of sub-specialization and QA at TMC. A quantitative evaluation of caseloads and feedback from secondary reviews was performed and reported to be exemplary for 2020/2021.

Some contents of this project report stemmed from the controlling department of TMC: continuous monitoring of caseloads, disagreement rates of secondary review and feedback, and external/independent review of cases. This report did not involve human subjects. Analysis was solely conducted among the co-authors of this report; thus, it was exempt from IRB review.

### 2.1. Qualitative Evaluation

The management of sub-specializations was explained in detail. The sequential character of QA at TMC was reported in a narrative fashion and illustrated in a flow diagram using Flowchart Designer 3.3.5 (Zhang Guangjian 2016–2019) for Mac.

### 2.2. (Semi-) Quantitative Evaluation

Statistical information about caseloads was extracted from the TMC RIS (Optemis, TMC, Barcelona, Spain) and visualized using Microsoft Excel 16.58 for Mac (Redmond, WA, USA).

Monitored disagreement rates of prospective secondary readings and feedback for 2021 were prepared for TMC’s elective sections and expressed as a percentage of all teleradiology reports. In comparison, the results of external audits of MSK and Neuro cases were provided.

## 3. Description of Sub-Specialization and Quality Assurance at TMC

### 3.1. Sub-Specialization at TMC

Sub-specialization at TMC is arranged by organ systems and is already a form of QA per se. Four experts in their fields are supervising as Head of Sections of musculoskeletal radiology (MSK), neuroradiology, head and neck, and the body section for elective cases. Emergency cases are read by another section. Every sub-specialized radiologist in each section reports on CR, CT, and MRI. QA is evaluated per section and allows for only narrow error margins; therefore, most radiologists meet this high-quality standard only when concentrating on a single section.

Radiologists must have worked in their specialty for at least two years after their board exam to be considered candidates to work for TMC. All TMC radiologists have the opportunity to increase their level of expertise in a narrower field of radiology and to pursue special interests. The TMC Academy was established to ensure continuing education via online and onsite learning opportunities and an inhouse developed reporting simulator. Additional financial support is available for external training.

The level of sub-specialization is monitored continuously on an individual basis as well. The section heads are aware of the strengths and weaknesses of their radiologist and can thus encourage improvement in certain “super sub-specializations”. According to the individual radiologists’ expertise profile, examinations are automatically assigned to the most qualified radiologist, i.e., with regard to dementia, the hip, or the pancreas.

### 3.2. Process of Quality Assurance at TMC

**Figure 1:**
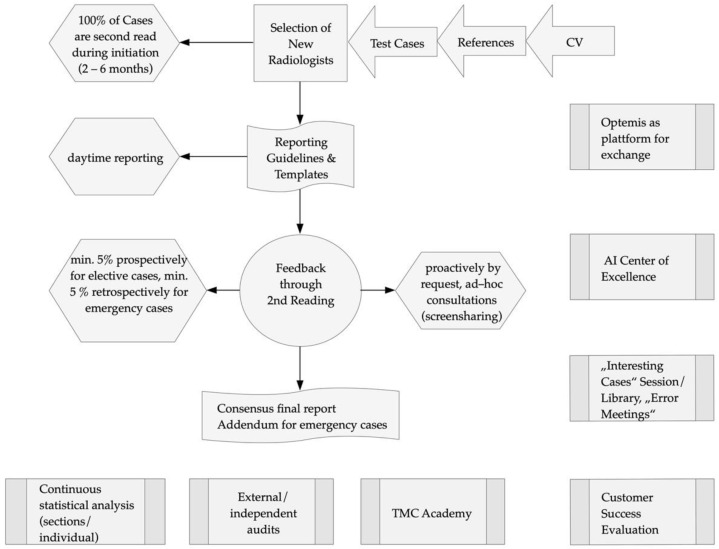
Quality assurance at TMC. Starting with the selection of a radiologist a complex system of QA contributes to a high quality of radiology reports.

The process of QA begins with the selection of new radiologists (Figure 1). Applicants are invited if their CV and references demonstrate a high level of sub-specialization. Through test cases, each section head determines the suitability for employment at TMC. In addition to excellent professional expertise, TMC demands the willingness and ability to receive and provide feedback continuously. Specific reporting guidelines and templates for structured reports are expected to be embraced during the onboarding process to foster reporting alignment as a key component to facilitate continuous peer-review and feedback processes for learning purposes. In addition, newly employed radiologists receive feedback through prospective secondary readings of all their radiology reports over a period of 2–6 months. As radiology reports are expected to be provided in the native language of TMC’s customers in Denmark, Sweden, and the UK, language teaching and editing can be part of the QA process depending on the individual language skills of radiologists.

A minimum of 5% of elective reports are randomly assigned to a second radiologist for peer review and feedback to the primary radiologist before the final report is delivered to the referring clinician. As a matter of course, radiologists are peer-reviewed only by the radiologists of their own department and field of expertise. All radiologists receive and provide peer-reviews alike after their individual training periods. It is believed that each feedback can be valuable despite differences in professional experience. This automated review and feedback process is unblinded and expedited by the inhouse developed RIS (Optemis, TMC, Barcelona, Spain). The second reader can feedback in full agreement with the primary report or can modify it when considered necessary and select a feedback option that reflects the clinical significance of the report modification. The clinical relevance of these discrepant image interpretations has been agreed to be the guiding criterion to provide feedback, to place the potential impact on patient management at the center of this process (Table 1, see Supplementary Figure S1 for Optemis screenshot). Reports with full agreement and clinically non-relevant report modifications are distributed to the referring clinician immediately after the secondary reading is completed. Reports with clinically relevant report modifications are not distributed but sent back for review to the first reader instead, who can either agree or disagree with the second reader. In case of agreement with the report modification, the new report version is distributed. If the first reader disagrees with the second reader, the two different report versions are sent to another radiologist, usually the corresponding head of the section, for evaluation and discrepancy resolution. It is mandatory to reach a consensus report for all discrepant cases between two radiologists through this arbitration process before the distribution of a final report version. For additional safety purposes in case of doubt about the clinical relevance of discrepant interpretations, second readers are encouraged to settle for the higher likelihood (“possibly clinically relevant” rather than “clinically not relevant”) to assure that a final consensus report is also reached related to the report modifications of rather doubtful clinical relevance. In addition to the random minimum of 5% of secondary readings, elective reporting radiologists can also voluntarily initiate a second reading process of any of their reports initially placed on a single reading pathway to receive peer feedback through a ”second opinion request” whenever they consider this to be justified based on their clinical judgment. For the emergency service the minimum peer review rate is lower, and the feedback is conducted retrospectively after the report has been delivered to the referring clinician. In case of a disagreement considered to be clinically relevant, an addendum is issued to the primary report, and the corresponding referring clinician is informed.

Feedback meetings are held weekly to share and discuss interesting cases. A selection of interesting cases is stored if permitted by the clients after anonymization for further teaching.

The structured big data generated out of the standardized peer feedback processes provide curated information to identify patterns of discrepancies and errors [33]. All non-conformities received from the client hospitals are registered and analyzed. In addition, external audits are scheduled annually to generate independent quality data and to identify additional errors. These are the three main sources of information used for the definition and implementation of the corresponding preventive actions to continuously lower the incidence of errors. Examples of relevant preventive actions that use these sources of quality information at TMC are the regularly held Radiology Events and Learning Meetings (REALM) and the recommendations given in the “*How to avoid common errors in Radiology*” document distributed to all TMC radiologists [34].

The Australian sites allow daytime reporting for emergency cases during the European night to reduce the risk of diagnostic errors due to fatigue [35]. Radiologists are supported by artificial intelligence (AI) algorithms during the detection of incidental pulmonary embolisms, intracranial hemorrhages, and lung nodules.

The quarterly evaluation of radiologists enables the identification of areas for individual improvement and the current level of expertise. Monitoring of reporting speed helps to identify possible causes for perception errors (false negatives). Continuous training of radiologists at the TMC academy is another component of QA at TMC.

During the implementation process of new customers, it is verified if the corresponding imaging protocols and images to be sent to TMC are at the expected levels of quality for interpretation and reporting purposes. Customer happiness is evaluated regularly to determine an average “Customer Effort Score” ranging from 1 (extremely difficult) to 7 (extremely easy), a “Net Promoter Score”, and an average “Customer Satisfaction Score” ranging from 1 (very dissatisfied) to 4 (very satisfied). An average “Helpfulness Score” evaluated the agreement with the statement “We give answers that help give care…?” ranging from 1 (strongly disagree) to 4 (strongly agree) (Table 2).

### 3.3. (Semi-) Quantitative Results

**Figure 2:**
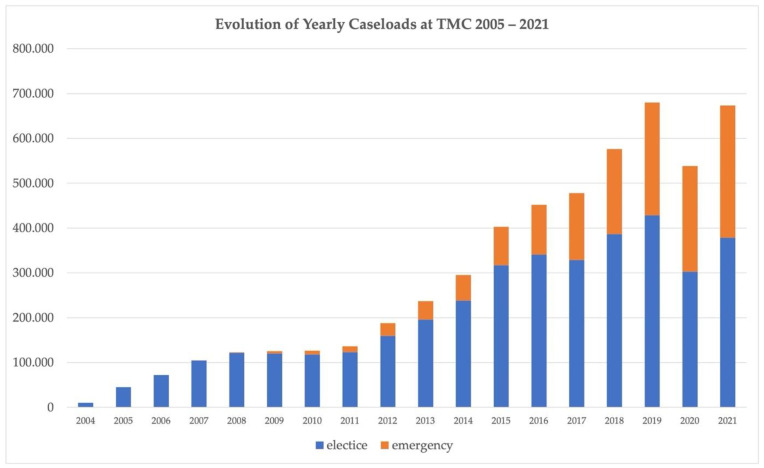
Evolution of caseload of elective and emergency teleradiology cases at TMC from 2005 to 2021.

Figure 2 illustrates the growth in case volumes reported by TMC radiologists from 2005 until 2021. After a slight drop in 2020 due to the COVID-19 pandemic, the yearly caseload stabilized and almost reached the level of 2019.

**Figure 3:**
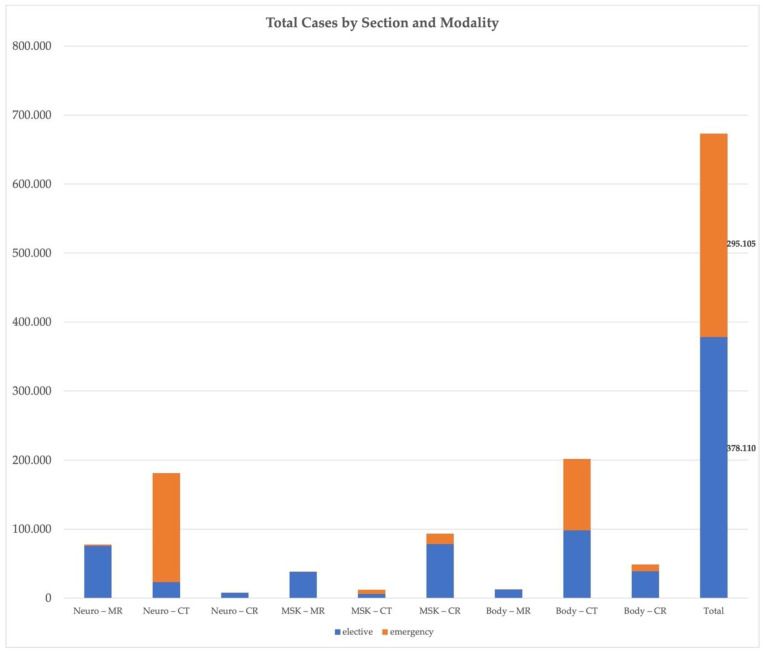
Total cases 2021. Total caseload of the elective (blue) and emergency (orange) teleradiology services provided by TMC in 2021 according to sub-specialization and modality.

Figure 3 illustrates the caseload for CR, CT, and MRI of every section for elective and emergency cases. A total of 673.215 cases were processed by TMC in 2021.

**Figure 4:**
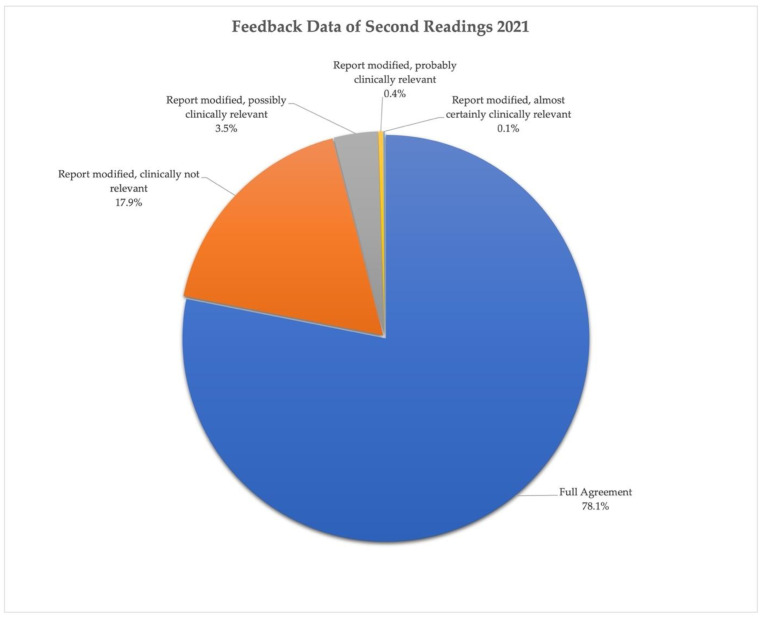
Feedback data of second readings for TMC’s elective sections in 2021.

The percentage of prospective secondary reviews for elective cross-sectional imaging was 11.9% for MSK, 7.4% for neuroradiology, and 5.5% for the body section in 2021 (overall 7.3%). A total of 2.6% of all emergency cases (CR, CT, and MRI) were reviewed retrospectively. The overall monitored feedback of prospective secondary readings in 2021 for all sections is shown in Figure 4 according to the type of feedback. The least possibly clinically relevant disagreement varied between 2.9% and 5.6% among the sections and was 4% for all prospective second readings. The results of external audits of randomly selected elective MSK and Neuro cases completed between 2018 and 2021 showed lower disagreement rates compared to the internal feedback data, with only 0–2% of the reports reviewed considered to contain mild inaccuracies with possibly clinically relevant consequences. The external audit outcome of all sections shows an overall lower discrepancy rate than internal feedback, including the clinically relevant discrepancies—the likely explanation for this being that the internal data are not pure audit data but influenced by the feedback and knowledge sharing requirement to allow the review of a modified report by the first reader even if the likelihood of clinical relevance is low. As commented in the previous section (Section 3.2) already, this type of feedback is encouraged by the section heads, thus increasing the number of “possibly” clinically relevant report modifications.

## 4. Discussion

The manuscript in hand describes sub-specialization and QA at one of the largest European teleradiology networks. Difficulties during the development of the company referred to technical and humane issues. Technological challenges were mastered through an individual approach and inhouse development. The company invested in an IT department that developed an individualized RIS and facilitated the integration of new customers. In a way, developments at TMC are similar to the development of noncommercial teleradiology networks worldwide [36,37,38]. Medical management and the IT department share the responsibility to decide about the introduction of a new AI product into a teleradiology service. Their decisions should be based on specific knowledge and internal evaluation processes [39]. TMC’s AI Center for Excellence addresses this need. Therefore, IT and software development may continue to play a supporting role in further technological development at TMC and the challenge of dealing with an increasing image load. AI can further enhance QA through the reduction of false negative findings [40] and support in handling growing image volumes through an efficiency increase in the reporting process.

Sub-specialization at TMC faces significant challenges in assuring fellow radiologists that they are appreciated and needed all over the world while convincing potential customers of the value of sub-specialization under the right circumstances. General radiologists still provide the vast majority of radiology care and in many clinical environments, sub-specialization is not possible due to technology restrictions or a lack of relevant case numbers. However, in large teleradiology networks, with a strong IT department and strong QA processes, sub-specialization can be delivered to smaller hospitals and increase report quality.

Additionally, the philosophy at TMC only expresses a general trend of specialization in medicine. Sub-specialized referring clinicians also expect sub-specialized radiology reports. To meet this demand, many general radiologists are already practicing at some sort of specialized level [41]. They need to be convinced that the concept of TMC provides opportunities for every colleague to enhance the pursuit of special interests [42]. The risk of turning into an isolated specialized expert should be met with frequent teamwork and participation at MDTMs. Current literature widely supports the superiority of sub-specialized radiology reports [43,44] and the incorporation of sub-specialized radiology can be regarded as a measure to meet sub-specialized clinical experts and contribute to improved clinical outcomes in patients [45]. Integration of platforms for inside and outside expert dialogues into the next generation of commercially available RIS/PACS environments will thus prove to be a major accelerator for sub-specialized teleradiology [26].

Moreover, the TMC staff are faced with the difficulty of persuading customers that sub-specialized radiology should be the standard of care rather than an exception. Not only complicated cases should be referred to a sub-specialized radiologist. Sub-specialization also improves the quality of presumably less complex cases, which represent the vast majority of the diagnostic imaging volume.

Through regular customer success surveys, sub-specialized radiologists can even adapt their reporting style to customer needs. Individual service approaches should come naturally to successful teleradiology providers who acknowledge the differing needs of medical facilities. Economic considerations are eminently influenced by TATs. A proven reduction in TATs could be another way to convince customers of sub-specialized teleradiology services such as TMC [16].

An important challenge of teleradiology services is the difficulty of establishing reliable partnerships through virtual channels. On the one hand, TMC is attempting to address this concern by grouping customers and radiology consultants to promote harmonization, personal relations, and trust. On the other hand, teleradiology services must strive to establish technical structures to improve obtaining feedback about patient outcomes in compliance with local General Data Protection Regulations (GDPR).

Promoting QA in the form of secondary readings is another challenge for modern teleradiology services such as TMC. Differences in measuring disagreement rates arise when attempting to compare the results of the literature; double-reporting, anonymous peer-review, and retrospective versus prospective secondary image interpretation influence the outcome of disagreement studies [26]. Nevertheless, the current literature supports the idea that peer-review should be part of self-critical radiology and QA [23,30,46]. Again, economic concerns play an important role—what is the economic optimum of quality? [47]. This question had to be addressed by TMC as well. The company estimated that only 1% of TMC’s overall costs are directly produced by secondary readings of 5% of all cross-sectional radiology reports—an amount which could appear as an acceptable if not necessary investment. In addition to bearing the costs for peer-review, investments in radiology leadership to oversee the implementation, maintenance, and execution of QA must be considered.

## 5. Conclusions

With continuing and current developments in radiology in mind, the essential features of sub-specialization and innovative quality assurance are relevant for teleradiology services and for most radiology departments and imaging networks worldwide in the future to respond to the increasing demand for value-based radiology.

## Figures and Tables

**Table 1:** Feedback options during second reading.

Feedback Options and Resulting Outcome	
Full Agreement	Report Distributed
Report modified, clinically not relevant	Report distributed
Report modified, possibly clinically relevant	Report not distributed and send back to first reader for review. Final consensus report required before distribution.
Report modified, probably clinically relevant	Report not distributed and send back to first reader for review. Final consensus report required before distribution.
Report modified, almost certainly clinically relevant	Report not distributed and send back to first reader for review. Final consensus report required before distribution.

**Table 2:** Factors and scoring of Customer Success Evaluation.

Customer Success Evaluation				
Factor	Customer Effort Score	Net Promoter Score	Customer Satisfaction Score	Helpfulness Score
**Scoring**	7 = extremely easy 6 = easy 5 = somehow easy 4 = neither difficult nor easy 3 = somehow difficult 2 = difficult 1 = extremely difficult	1–10: 9 of 10 = Promotor 7 or 8 = Passives 0–6 = Detractor	4 = very satisfied 3 = satisfied 2 = dissatisfied 1 = very dissatisfied	4 = strongly agree 3 = agree 2 = disagree 1 = strongly disagree

## Data Availability

Data are contained within the article or Supplementary Material.

## Supplementary Materials

The following supporting information can be downloaded at: https://www.mdpi.com/article/10.3390/healthcare10061001/s1, Supplementary Figure S1: Custom made TMC RIS (Optemis, TMC, Barcelona).
