# Supplementary material for: Quality Assurance of a Cross-Border and Sub-Specialized Teleradiology Service
Source: Healthcare (Basel). 2022 May 28;10(6):1001. doi: 10.3390/healthcare10061001 (PMC9223114; doi:10.3390/healthcare10061001)
Supplement: Supplementary file 1 [file healthcare-10-01001-s001.zip › healthcare-1687485-supplementary.pdf]

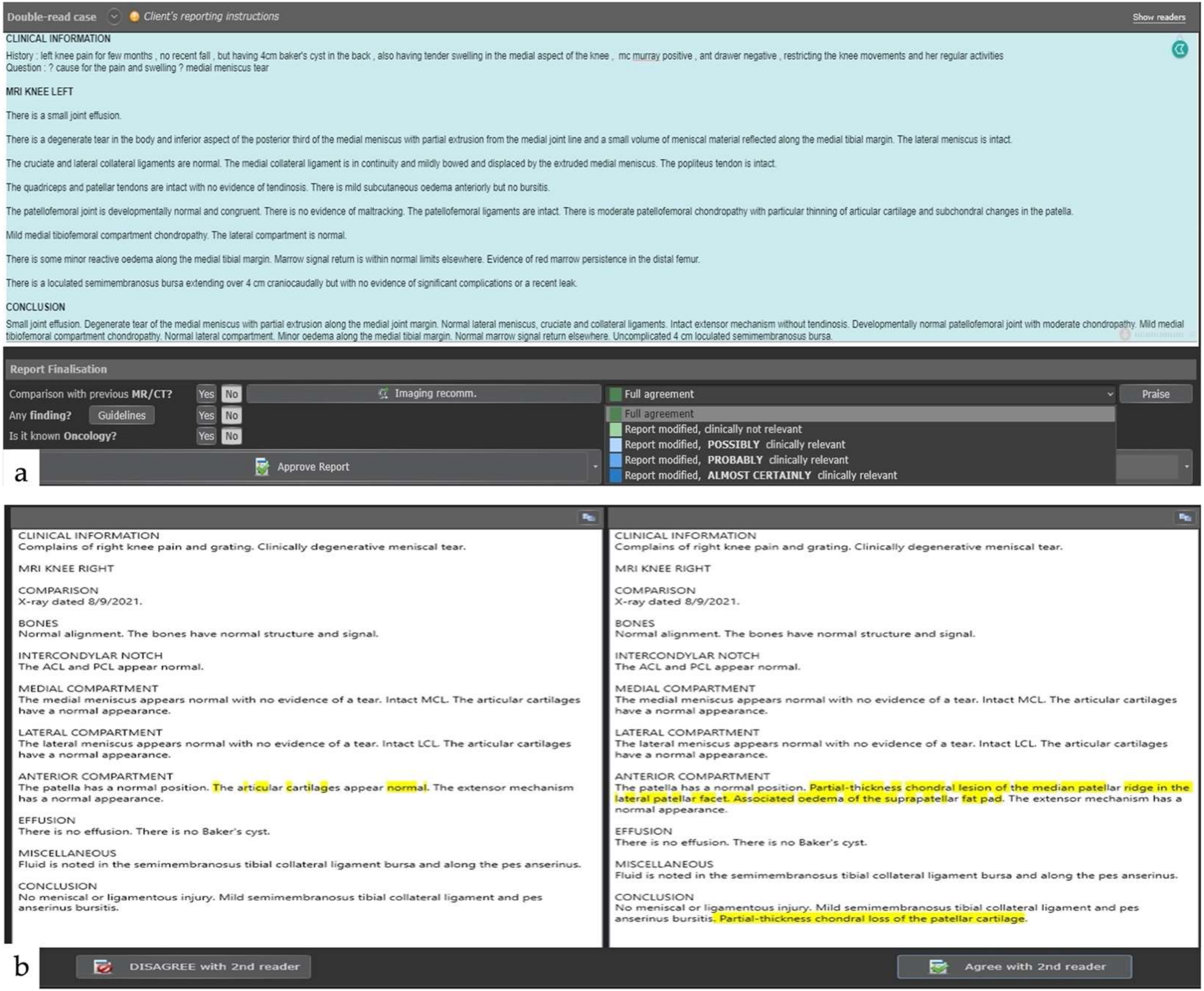
Double-read case
Client's reporting instructions
Show readers
CLINICAL INFORMATION
History : left knee pain for few months , no recent fall , but having 4cm baker's cyst in the back , also having tender swelling in the medial aspect of the knee , mc murray positive , ant drawer negative , restricting the knee movements and her regular activities
Question : ? cause for the pain and swelling ? medial meniscus tear
MRI KNEE LEFT
There is a small joint effusion.
There is a degenerate tear in the body and inferior aspect of the posterior third of the medial meniscus with partial extrusion from the medial joint line and a small volume of meniscal material reflected along the medial tibial margin. The lateral meniscus is intact.
The cruciate and lateral collateral ligaments are normal. The medial collateral ligament is in continuity and mildly bowed and displaced by the extruded medial meniscus. The popliteus tendon is intact.
The quadriceps and patellar tendons are intact with no evidence of tendinosis. There is mild subcutaneous oedema anteriorly but no bursitis.
The patellofemoral joint is developmentally normal and congruent. There is no evidence of maltracking. The patellofemoral ligaments are intact. There is moderate patellofemoral chondropathy with particular thinning of articular cartilage and subchondral changes in the patella.
Mild medial tibiofemoral compartment chondropathy. The lateral compartment is normal.
There is some minor reactive oedema along the medial tibial margin. Marrow signal return is within normal limits elsewhere. Evidence of red marrow persistence in the distal femur.
There is a loculated semimembranosus bursa extending over 4 cm craniocaudally but with no evidence of significant complications or a recent leak.
CONCLUSION
Small joint effusion. Degenerate tear of the medial meniscus with partial extrusion along the medial joint margin. Normal lateral meniscus, cruciate and collateral ligaments. Intact extensor mechanism without tendinosis. Developmentally normal patellofemoral joint with moderate chondropathy. Mild medial tibiofemoral compartment chondropathy. Normal lateral compartment. Minor oedema along the medial tibial margin. Normal marrow signal return elsewhere. Uncomplicated 4 cm loculated semimembranosus bursa.
Report Finalisation
Comparison with previous MR/CT?
Yes
No
Imaging recomm.
Full agreement
Praise
Any finding?
Guidelines
Yes
No
Is it known Oncology?
Yes
No
Full agreement
Report modified, clinically not relevant
Report modified, POSSIBLY clinically relevant
Report modified, PROBABLY clinically relevant
Report modified, ALMOST CERTAINLY clinically relevant
Approve Report
a
CLINICAL INFORMATION
Complains of right knee pain and grating. Clinically degenerative meniscal tear.
MRI KNEE RIGHT
COMPARISON
X-ray dated 8/9/2021.
BONES
Normal alignment. The bones have normal structure and signal.
INTERCONDYLAR NOTCH
The ACL and PCL appear normal.
MEDIAL COMPARTMENT
The medial meniscus appears normal with no evidence of a tear. Intact MCL. The articular cartilages have a normal appearance.
LATERAL COMPARTMENT
The lateral meniscus appears normal with no evidence of a tear. Intact LCL. The articular cartilages have a normal appearance.
ANTERIOR COMPARTMENT
The patella has a normal position. The articular cartilages appear normal. The extensor mechanism has a normal appearance.
EFFUSION
There is no effusion. There is no Baker's cyst.
MISCELLANEOUS
Fluid is noted in the semimembranosus tibial collateral ligament bursa and along the pes anserinus.
CONCLUSION
No meniscal or ligamentous injury. Mild semimembranosus tibial collateral ligament and pes anserinus bursitis.
CLINICAL INFORMATION
Complains of right knee pain and grating. Clinically degenerative meniscal tear.
MRI KNEE RIGHT
COMPARISON
X-ray dated 8/9/2021.
BONES
Normal alignment. The bones have normal structure and signal.
INTERCONDYLAR NOTCH
The ACL and PCL appear normal.
MEDIAL COMPARTMENT
The medial meniscus appears normal with no evidence of a tear. Intact MCL. The articular cartilages have a normal appearance.
LATERAL COMPARTMENT
The lateral meniscus appears normal with no evidence of a tear. Intact LCL. The articular cartilages have a normal appearance.
ANTERIOR COMPARTMENT
The patella has a normal position. Partial-thickness chondral lesion of the median patellar ridge in the lateral patellar facet. Associated oedema of the suprapatellar fat pad. The extensor mechanism has a normal appearance.
EFFUSION
There is no effusion. There is no Baker's cyst.
MISCELLANEOUS
Fluid is noted in the semimembranosus tibial collateral ligament bursa and along the pes anserinus.
CONCLUSION
No meniscal or ligamentous injury. Mild semimembranosus tibial collateral ligament and pes anserinus bursitis. Partial-thickness chondral loss of the patellar cartilage.
b
DISAGREE with 2nd reader
Agree with 2nd reader

Figure S1. Custom made TMC RIS (Optemis, TMC, Barcelona). (a) In the lower right corner, the levels of disagreement with the primary report (shaded in light blue) can be selected be the second reader. (b) The primary reader receives a feedback message and the modified report with highlighted (yellow) changes. The primary reader can either disagree with the changes or agree to the consensus report. In case of disagreement a third radiologist involved seeking an agreement.
